# Transcranial direct current stimulation for the treatment of tinnitus: a review of clinical trials and mechanisms of action

**DOI:** 10.1186/s12868-018-0467-3

**Published:** 2018-10-25

**Authors:** Tifei Yuan, Ali Yadollahpour, Julio Salgado-Ramírez, Daniel Robles-Camarillo, Rocío Ortega-Palacios

**Affiliations:** 1 Shanghai Key Laboratory of Psychotic Disorders, Shanghai Mental Health Center, Shanghai Jiao Tong University School of Medicine, Shanghai, China; 2 Co-innovation Center of Neuroregeneration, Nantong University, Nantong, Jiangsu China; 3 Department of Medical Physics, School of Medicine, Ahvaz Jundishapur University of Medical Sciences, Golestan Blvd, Ahvaz, 61357-33118 Iran; 4 Biomedical Engineering Department, Polytechnic University of Pachuca, Zempoala, Mexico; 5 Graduate and Research Department, Polytechnic University of Pachuca, Zempoala, Mexico

**Keywords:** Transcranial direct current stimulation, Tinnitus, Treatment, Clinical efficacy

## Abstract

**Background:**

Tinnitus is the perception of sound in the absence of any external acoustic stimulation. Transcranial direct current stimulation (tDCS) has shown promising though heterogeneous therapeutic outcomes for tinnitus. The present study aims to review the recent advances in applications of tDCS for tinnitus treatment. In addition, the clinical efficacy and main mechanisms of action of tDCS on suppressing tinnitus are discussed.

**Methods:**

The study was performed in accordance with the PRISMA guidelines. The databases of the PubMed (1980–2018), Embase (1980–2018), PsycINFO (1850–2018), CINAHL, Web of Science, BIOSIS Previews (1990–2018), Cambridge Scientific Abstracts (1990–2018), and google scholar (1980–2018) using the set search terms. The date of the most recent search was 20 May, 2018. The randomized controlled trials that have assessed at least one therapeutic outcome measured before and after tDCS intervention were included in the final analysis.

**Results:**

Different tDCS protocols were used for tinnitus ranging single to repeated sessions (up to 10) consisting of daily single session of 15 to 20-min and current intensities ranging 1–2 mA. Dorsolateral prefrontal cortex (DLPFC) and auditory cortex are the main targets of stimulation. Both single and repeated sessions showed moderate to significant treatment effects on tinnitus symptoms. In addition to improvements in tinnitus symptoms, the tDCS interventions particularly bifrontal DLPFC showed beneficial outcomes on depression and anxiety comorbid with tinnitus. Heterogeneities in the type of tinnitus, tDCS devices, protocols, and site of stimulation made the systematic reviews of the literature difficult. However, the current evidence shows that tDCS can be developed as an adjunct or complementary treatment for intractable tinnitus. TDCS may be a safe and cost-effective treatment for tinnitus in the short-term application.

**Conclusions:**

The current literature shows moderate to significant therapeutic efficacy of tDCS on tinnitus symptoms. Further randomized placebo-controlled double-blind trials with large sample sizes are needed to reach a definitive conclusion on the efficacy of tDCS for tinnitus. Future studies should further focus on developing efficient disease- and patient-specific protocols.

## Background

Tinnitus is the perception of sound, in the ear or in the head, in the absence of any external acoustic stimulation which affects 10–15% of the adult population worldwide [1, 2]. The main risk factors of tinnitus include hearing loss, trauma to the auditory periphery such as a lesion to auditory nerve, abnormal plastic changes in auditory network, ototoxic medications, head injury, and depression [3]. Hearing loss is not necessarily a precondition of tinnitus; however, some studies have shown that different forms of hearing loss may have correlation with tinnitus [2, 4]. This disorder is usually accompanied by different mild to severe comorbidities such as depression, anxiety, and sleep disturbances that make it a debilitating condition [1, 2, 5].

Neuroimaging, neuroelectrophysiologic, and neuroanatomic studies have shown that maladaptive plastic changes in different auditory and non-auditory cerebral regions and abnormal neural activities of specific cortical regions might be the main etiology of tinnitus [6–10]. Tinnitus perception is an integrated output of a large and complicated brain network comprising of different subnetworks with overlapping functions [2, 11]. In this impaired network, each subnetwork represents a clinical aspect of tinnitus such as distress, loudness, and laterality [11–13]. Neurobiological and neuroimaging findings have shown that abnormal activities of the non-auditory regions associated with cognitive and attentional functions as well as limbic processes probably contribute to the unpleasant and distressing aspects of tinnitus [11, 14]. Structural and functional abnormalities in dorsolateral prefrontal cortex (DLPFC) [10, 15–17] and auditory cortex (AC) [2, 3, 18, 19] are associated with tinnitus. The DLPFC is a multifunction region that plays important roles in auditory processing and perception, auditory attention, top-down modulation of auditory processing, and modulating the input to primary AC [20–22]. Moreover, DLPFC is involved in regulating different cognitive functions. Therefore, in development of any treatment modality for tinnitus, this disorder should be considered as a complex and heterogamous condition involving a large network consisting of multiple overlapping brain networks. Considering the engagements of AC and DLPFC in the tinnitus perception, these regions may be good targets for any therapeutic intervention for tinnitus.

Several pharmacologic agents have been developed for tinnitus treatment; however, a large portion of the patients are resistant to the treatment [1]. In addition, most of the pharmacologic drugs are associated with different side effects that adversely influence the individual’s daily and quality of life. So far, no definitive treatment has been proposed for tinnitus and several common causes of tinnitus remain elusive. In this regard, studies are ongoing to develop new efficient therapeutic modalities for tinnitus in two avenues including pharmacologic agents and non-pharmacologic modalities.

During the recent decades, several non-pharmacological modalities such as cognitive behavioral therapies [23], noise-masking modality [24], and neurofeedback [25] have been proposed for treatment of tinnitus; however, they have limited treatment efficacy and each of them have their own drawbacks.

Applications of brain stimulation and modulation techniques have been dramatically developed during the recent decade for treatment and management of neuropsychiatric disorders [26–31]. These modalities including repetitive transcranial magnetic stimulation (rTMS), deep brain stimulation, and electrical stimulation have shown promising outcomes in the disorders in which the abnormal neural activities and impaired neural interfaces are the main characteristics [26–30]. This significant contribution of neural stimulation and modulation modalities is mostly because of mutual interactions between the endogenous and exogenous electrical and magnetic fields. The therapeutic values of electric and magnetic fields have been reported in different disorders that support the above claim [32–35].

RTMS has shown therapeutic effects in tinnitus through eliminating the tinnitus symptoms and also improving the cognitive impairments comorbid with tinnitus [36–38]. However, this technique is relatively expensive and associated with side effects with lower mobility.

Transcranial direct current stimulation (tDCS) is a form of neuromodulation in which a low intensity direct current passes the brain tissues through a pair of electrodes placed on the scalp. The tDCS is a noninvasive, safe, cost-effective, and user friendly modality which has shown promising outcomes in treatment of different neuropsychiatric disorders as well as in improving cognitive functions in healthy individuals [39–43].

Tinnitus is associated by abnormal neural activities in different brain regions and also maladaptive neuroplasticity of specific regions. Therefore, tDCS applied over specific brain regions with appropriate anodal/cathodal placement has been expected to have beneficial effects for this disorder. Anodal and cathodal tDCS respectively increases (depolarizes) and reduces (hyperpolarizes) the cortical excitability of the exposed regions [44].

Several preclinical and clinical studies have been conducted on tinnitus and the initial findings were promising though controversial [45–48]. Studies are ongoing to reach a definitive answer on the clinical efficacy of tDCS in tinnitus.

Studies are ongoing to develop effective clinical protocols and to understand the mechanisms of action. The present study aims to review the recent advances in applications of tDCS for tinnitus treatment. In addition, the clinical efficacy and the main mechanisms of action of the technique are discussed.

## Methods

The databases of the PubMed (1980–2018), Embase (1980–2018), PsycINFO (1850 –2018), CINAHL, Web of Science, BIOSIS Previews (1990–2018), Cambridge Scientific Abstracts (1990–2018), and google scholar (1980–2018) using the set search terms. The study procedures were performed according to the guidelines of the PRISMA. The search terms were “transcranial direct current stimulation” OR “tDCS” AND “tinnitus” AND “treatment”. The date of the most recent search was 20 May 2018. Bibliographies of the retrieved records and review articles were manually reviewed to identify the records that may have been missed in the initial search. The titles, abstracts, and keywords of all retrieved records were reviewed and the eligible records were entered in the final review based on the inclusion and exclusion criteria. Only published, peer-reviewed studies on human subjects available in English were considered for this review. The studies that investigated the treatment of different types of tinnitus with different tDCS devices and in different protocols against sham condition were included in the review. Studies of randomized controlled trials were included.

The studies should have assessed at least one therapeutic outcome measured before and after an intervention. The studies that assessed only cognitive measures, studies on animal and healthy subjects were also excluded. Clinical trials without a randomized controlled design, conference abstracts, narrative reviews, and editorials were excluded from the review.

## Results

**Fig. 1:**
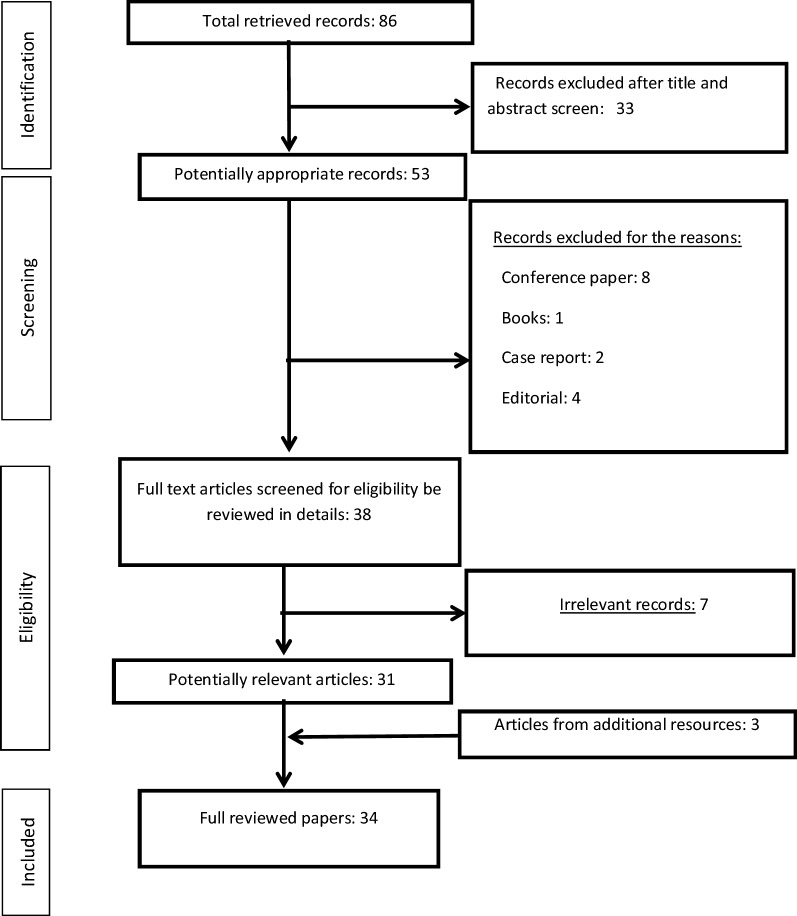
The PRISMA flow chart of the study

A total of 85 studies were identified at the screening step. In the identification phase, total of 33 records were excluded from the further assessment and 53 records were entered into the screening phase in which 8 conference abstracts, 1 book, 2 case reports and 4 editorials were excluded from the review. In the eligibility stage, 31 records remained in the study and 3 records from the additional records were added into the study where total of 34 studies were included for detailed review. Due to the heterogeneities in the patients and the tDCS devices and protocols as well as the target sides, the authors decided to comprehensively review the studies. The review focuses on the advances in applications of tDCS for treatment of tinnitus and the important factors in the resulting outcomes. In addition, the mechanisms of actions of the tDCS in tinnitus treatment are discussed (Fig. 1).

## Discussion

Tinnitus is a heterogeneous disorder in which several regions are involved in the tinnitus-related anomalies ranging primary and secondary auditory systems as well as non-auditory brain areas.

The general hypothesis in application of tDCS for treatment of tinnitus like other neuropsychiatric disorders is that anodal tDCS increases the neural excitability, whereas cathodal tDCS decreases it. As a result: anodal tDCS with excitatory effect can be applied on the regions with hypo-activity associated with an impairment to reach beneficial outcome. Similarly, cathodal tDCS that induces inhibitory effect can be applied over the regions with disease specific hyper-activities to reach beneficial effects.

The main approach in choosing the target site and electrode placement in tDCS applications in different neuropsychiatric disorders is modulating the impaired region(s) of the brain to alter the activities or functions of the region(s) towards normal conditions. In this regard, for tinnitus, the main objective is modulating either the tinnitus percept or its affective aspects like distress through disrupting the underlying pathological neural activities. The general hypothesis is that anodal tDCS increases the neural excitability, whereas cathodal tDCS decreases it [44, 49–51]. As a result: anodal tDCS with excitatory effect can be applied on the regions with hypo-activity associated with an impairment to reach beneficial outcome [52]. Similarly, cathodal tDCS that induces inhibitory effect can be applied over the regions with disease specific hyper-activities to reach beneficial effects [52].

Considering the tinnitus features and the associations between tinnitus and the structural and functional abnormalities in DLPFC [10, 15–17] and AC [2, 3, 18, 19], these two sites were the main targets in the previous tDCS studies in tinnitus. Initial tDCS studies have targeted the AC for tinnitus treatment and the findings were promising though controversial [53–55]. Moreover, modulating DLPFC activity using tDCS has been shown to enhance different cognitive functions in healthy individual and to improve different neuropsychiatric disorders, including tinnitus [17, 39, 40, 56–58].

The two main sites targeted in the previous studies for treatment of tinnitus were DLPFC [13, 59, 60] and AC [45, 54, 61]. Fregni et al. conducted the first study investigating tDCS in tinnitus in which they compared the effects of cathodal tDCS, anodal tDCS, and 10-Hz rTMS against sham stimulation over two sites of mesial parietal cortex and left temporoparietal area (LTA) on tinnitus symptoms [45]. They reported that 10 Hz rTMS and anodal tDCS of LTA significantly reduced the tinnitus symptoms; however, the effect was transient and short lasting. After this study, several clinical trials have been conducted to evaluate and develop the therapeutic efficacy of tDCS for treatment of tinnitus. Garin et al. investigated the outcomes of tDCS applied over LTA and reported significant improvements in tinnitus symptoms and interestingly they reported the beneficial effects lasted for several days in some patients [62]. Both Fregni et al. and Garin et al. reported that cathodal tDCS over the LTA with the anode on the contralateral supraorbital area did not improve tinnitus symptoms.

Following these initial studies, other researchers have investigated the tDCS in single and repeated sessions over LTA and AC and reported controversial findings [54, 63, 64]. It seems that cathodal tDCS in single session is not effective on tinnitus treatment since cathodal tDCS is not strong enough to disturb or modulate the ongoing tinnitus-related abnormal cortical activities [65]. In this regard, repeated sessions of cathodal tDCS may have therapeutic effects on tinnitus based on the theoretical and experimental results. Therefore, repeated sessions of tDCS, longer period of each session and higher intensities have been designed to investigate the effects of cathodal tDCS in tinnitus [54, 60, 66, 67].

Following the initial studies focusing on temporoparietal area (TA) and AC, several studies have targeted prefrontal cortex (PFC), particularly DLPFC for tinnitus treatment. In these studies, the main target site was DLPFC and the most frequent electrode montage was bifrontal [13, 46, 59, 68]. Vanneste et al. were the first group reported the effects of bifrontal tDCS on tinnitus symptoms [13]. They investigated the effects of bifrontal tDCS over DLPFC (n = 478) in an open label study. They applied bifrontal tDCS (2 mA, each session 20 min) in two montages (anode right/cathode left (n = 448) and anode left/cathode right (n = 30) DLPFC) for 20 min. They reported no tinnitus-suppressing effect in the anode left/cathode right DLPFC tDCS. However, anode right/cathode left tDCS significantly reduced the tinnitus intensity or distress in 29.9% of the patients. In addition, they observed an interaction between the amount of distress reduction and the tinnitus laterality. However, they did not observe such interaction for the tinnitus intensity [13]. Vanneste et al. concluded that bifrontal tDCS could modulate the emotional aspects of tinnitus experienced by the patients [13].

The next studies conducted on bifrontal tDCS have reported that this montage both in anode left/cathode right or vice versa could improve the tinnitus-related depression and anxiety, respectively [47]. These effects could be attributed the roles of PFC and particularly DLPFC in modulating different non-auditory structures and networks involved in perception of the auditory and distress aspects of tinnitus as well as emotional functions.

To improve the tDCS protocol in targeting the optimal stimulation site for tinnitus treatment, De Ridder and Vanneste (n = 675) compared the efficacy of EEG-driven tDCS versus standard bifrontal tDCS [61]. They used source localized resting-state electrical activity to determine gamma-band functional connectivity as an index of the tinnitus network. On the one hand, the authors reported that standard bifrontal tDCS, with the anode right/cathode left DLPFC, significantly reduced tinnitus symptoms in 30% of the patients [61]. Moreover, the EEG-driven tDCS approach did not significantly improve the symptoms. The authors also tried to identify the mechanism of action of tDCS in suppressing the tinnitus symptoms through comparing the pre- and post-intervention of the source-localized resting-state electrical activity of the patients. They concluded that the tDCS induced changes are probably occurred through modulations of a large network consisting of pregenual anterior cingulate cortex, parahippocampal area, and right primary AC in resting-state spontaneous brain activity. This study demonstrated that tDCS impacts both the direct target under the electrodes (DLPFC) and distant regions with functional connections with the exposed target [61]. This finding along with neuroimaging studies encourage further studies on the therapeutic outcomes of tDCS applied over non-auditory regions in tinnitus.

In line with the optimization of tDCS protocol for tinnitus, Shekhawat and Vanneste designed a trial to optimize the parameters of bifrontal tDCS over DLPFC for tinnitus suppression with the primary outcome of tinnitus loudness [68]. They designed a dose–response trial (n = 111) to optimize the current intensity (1.5 and 2 mA), stimulation duration (20 and 30 min), and number of tDCS sessions (2, 4, 6, 8, and 10 with 3–4 day washout period between each session). The patients received a minimum of 2 sessions during 1 week or maximum of 10 sessions during 5 weeks. Their findings showed a significant reduction in tinnitus loudness after DLPFC tDCS. The intensity and duration of each session did not show significant interaction with the outcome. In addition, they reported that increasing the number of sessions increases the amount of outcome, but after 6 sessions no further increase was observed and the amount of outcome reached a plateau trend [68].

Few studies have used different electrode montages than the previous studies triggering LTA or AC and DLPFC. For instance Pal et al. in a randomized, parallel, double-blind, sham-controlled study investigated the treatment efficacy and safety of cathodal tDCS to the AC with anode over the PFC [69]. They applied a 5-session tDCS over five consecutive days and assessed the tinnitus handicap inventory (THI) score as the primary outcome of tinnitus after the last session on day 5, and at 1 and 3 months post stimulation. They reported no beneficial effects of tDCS on the neither primary nor secondary outcome measures. Their findings showed that tDCS of the auditory and prefrontal cortices does not improve tinnitus but it is relatively safe protocol [69].

A line of studies have focused on combinations of tDCS with other treatment modalities including pharmacological and non-pharmacological modalities [63, 70]. Shekhawat et al. in a 7-month long double-blind randomized clinical trial investigated the effects of multisession anodal tDCS over LTA combined with the hearing aid sound therapy in patients with chronic tinnitus (n = 40). They applied anodal tDCS (2 mA intensity; 20-min duration) for 5 consecutive sessions with 24-h gap over the LTA, and then applied a hearing aid treatment for 6 months. Their findings showed a significant improvement in the overall Tinnitus Functional Index score as well as the tinnitus loudness and distress scores. They reported that after 3 months of hearing aid use, significant improvements were observed in tinnitus that were sustained at 6 months of use [63].

Teismann et al. investigated the effects of combined tailor-made notched music training (TMNMT) with tDCS on tinnitus symptoms. They applied TMNMT for 10 subsequent days (daily single session of 2.5 h). During the initial 30-min of the first 5 days of the TMNMT sessions, they concurrently applied tDCS (intensity: 2 mA) in anodal, cathodal, and sham groups. The active electrode was over the head surface over left AC; the reference electrode was put over right supraorbital cortex [70]. They observed a significant reduction in tinnitus handicap inventory (THI) score that reached its maximum value after the 5 days of treatment. The treatment effect remained significant for 31 days following the termination of the treatment. They also reported no significant difference between the anodal, cathodal, or sham tDCS groups.

It seems that tDCS over TA or AC may have greater therapeutic effects when combined with other non-medication modalities.

So far, most of the tDCS trials for tinnitus treatment have investigated the effects of single session tDCS on tinnitus symptoms. However, few studies have assessed the treatment effects of repeated sessions of tDCS on tinnitus symptoms. In these studies the number of total sessions ranged three to ten sessions consisting daily one session and current intensity ranging 1–2 mA and each session lasting 15 to 30 min. The main target sites in the tDCS applications in tinnitus treatment were temporal or temporoparietal (auditory) cortex [48, 54, 71, 72] and DLPFC [47, 55, 73]. The findings of these studies are promising though heterogeneous which encourage conducting further placebo-controlled randomized trials to shed more light on the clinical efficacy of the technique and mechanism of action involved in the effects. One important factor that should be further assessed in future studies is assessing the treatment outcomes for longer follow up periods since most of the previous studies have investigated the transient effects of tDCS and in few cases the after effect assessments were not beyond some hours.

Neuroimaging and neurobiological studies have demonstrated that the main features of tinnitus are hyperactivity and maladaptive plasticity in AC [2, 6, 9]. In tinnitus there are specific neural changes that start at the cochlear nucleus and project to the AC and non-auditory brain regions. The main cause of these neural anomalies is maladaptive neural plasticity. This maladaptive plasticity increases spontaneous firing rates of and synchrony among neurons in primary and secondary auditory systems that may generate the phantom percept. In addition to the abnormal neural activities and maladaptive plasticity present in the primary and secondary ACs, disturbances in non-auditory brain structures and networks such as the insula, anterior cingulate cortex, and the DLPFC have been proposed as other possible pathologies of tinnitus [6, 8–10, 13, 18, 74, 75]. The perception of tinnitus has been reportedly as an integrated output of a complex tinnitus network consisting of different regions and subnetworks. It is assumed that each subnetwork of this network represents a clinical aspect of tinnitus such as distress, loudness, laterality, etc.

There are different hypotheses proposed to explain the therapeutic outcomes of tDCS in tinnitus symptoms. The first hypothesis is based on the disturbing theory of an ongoing neural activity associated with tinnitus. It is hypothesized that tDCS disturbs the abnormal ongoing neural activity induced by tinnitus. The second hypothesis is changing the maladaptive plasticity of tinnitus through repeated sessions of tDCS. Previous studies have shown that repeated sessions of tDCS depending on the polarity of the electrode could reduce or increase the neural excitability of the exposed regions and the resulting changes persist beyond the tDCS intervention [44, 76]. This altered excitability can lead to neuroplasticity with therapeutic effects for tinnitus [52]. Therefore, in the treatment of tinnitus with tDCS, the main idea is modulating the abnormal excitability in the auditory pathways and maladaptive plasticity in auditory and limbic cortexes through applying single or repeated sessions of tDCS. The clinical trials conducted so far have shown that single and repeated sessions of tDCS applied over DLPFC or AC may induce transient and long lasting therapeutic effects in tinnitus patients. The early studies have investigated the effects of single session tDCS and reported transient beneficial effect, but the effects did not last more than some hours.

Some evidence showed that the tDCS effects on tinnitus symptoms are probably induced through modulations of a large neural network comprising of pregenual anterior cingulate cortex, parahippocampal area, and right primary AC in resting-state spontaneous brain activity [61]. According to this hypothesis, the tDCS influences both the direct target under the electrodes and distant regions with functional connections with the direct target [61].

Most of the previous studies have investigated the physical parameters of tDCS to develop effective treatment protocols for tinnitus and also in other neuropsychiatric disorders including the electrode size, polarity, electrode placement and configuration, current amplitude and density, treatment duration, number of sessions and total dose. Findings of the recent studies as well as neuroimaging and neuroelectrophysiologic studies showed that tinnitus is a heterogeneous disease with different disease-specific features. It seems that in addition to the physical parameters of tDCS, the patient- and disease-specific factors including gender, audiometric variables, severity of tinnitus, tinnitus laterality and type, illness duration, and audiometric features of the patients might be important in exerting and/or the amount of therapeutic effects [47, 59, 60]. Therefore, at least one line of the future tDCS studies for treatment of tinnitus should focus on developing disease specific of tDCS protocols.

## Conclusions

This study reviewed the advances in using tDCS for treatment of tinnitus and discussed the therapeutic efficacy of the technique and the main mechanisms of action in treatment of tinnitus symptoms and therapeutic effects. Reviewing the current clinical trials showed that tDCS has moderate and promising treatment outcomes in the treatment of tinnitus. In addition, tDCS has shown beneficial effects on different cognitive impairments comorbid with tinnitus including anxiety and depression. However, so far there is no standard tDCS protocol for tinnitus treatment for clinical applications.

The main limitations of the conducted trials are small sample size, heterogeneities in patients and treatment protocols, poor methodology design, as well as the heterogeneous nature of tinnitus.

To develop efficient tDCS protocols for tinnitus, the roles of specific features of patient and tinnitus such as audiometric features of the patients, tinnitus laterality, tinnitus type, and tinnitus duration should be evaluated as well as the effects of the stimulation parameters. Further prospective, randomized, placebo-controlled, double-blind studies with large sample sizes are needed to reach a definitive conclusion on the efficacy of tDCS for tinnitus patients. Future studies should focus on developing efficient disease- and patient-specific protocols.

## Abbreviations

AC: auditory cortex
LTA: left temporoparietal area
PFC: prefrontal cortex
PRISMA: preferred reporting items for systematic reviews and meta-analyses
rTMS: repetitive transcranial magnetic stimulation
TA: temporoparietal area
tDCS: transcranial direct current stimulation
THI: tinnitus handicap inventory
TMNMT: tailor-made notched music training
